# Functional insights of plant bcl-2–associated ahanogene (BAG) proteins: Multi-taskers in diverse cellular signal transduction pathways

**DOI:** 10.3389/fpls.2023.1136873

**Published:** 2023-03-28

**Authors:** Hailong Jiang, Xiaoya Liu, Peixiang Xiao, Yan Wang, Qihui Xie, Xiaoxia Wu, Haidong Ding

**Affiliations:** ^1^College of Bioscience and Biotechnology, Yangzhou University, Yangzhou, China; ^2^Joint International Research Laboratory of Agriculture and Agri-Product Safety of Ministry of Education of China, Yangzhou, China

**Keywords:** abiotic and biotic stress, Bcl-2-associated athanogene (BAG), functional mechanism, plant development, protein structure

## Abstract

Bcl-2-associated athanogene (BAG) gene family is a highly conserved molecular chaperone cofactor in evolution from yeast to humans and plants playing important roles in a variety of signal pathways. Plant BAG proteins have special structures, especially those containing CaM-binding IQ motifs which are unique to plants. While early studies focused more on the structure and physiological function of plant BAGs, recent studies have revealed many novel functional mechanisms involved in multiple cellular processes. How to achieve signal specificity has become an interesting topic of plant BAG research. In this review, we have provided a historic view of plant BAG research and summarized recent advances in the establishment of BAG as essential components in normal plant growth, environmental stress response, and plant immunity. Based on the relationship between BAG proteins and their newly interacting proteins, this review highlights the functional mechanisms of various cellular signals mediated by plant BAGs. Future work needs to focus on the post-translational modification of BAG proteins, and on understanding how specificity is achieved among BAG signaling pathways.

## Introduction

Bcl-2-associated athanogene (BAG) gene family is a highly conserved molecular chaperone cofactor in evolution from yeast to humans and plants. BAG protein was first found in animals. As early as 1995, protein interaction cloning technology was used to screen mouse embryonic cDNA library using human B-cellymphoma-2 (Bcl-2) protein as bait protein and the first BAG-1 was found (Takayama et al., 1995). Since then, the biological function of BAGs has been widely investigated. There are six BAG members found in the human genome. The evolutionarily conserved BAG domain at the C-terminal end enables the protein family to bind to HSP70 related proteins, while different domains at the N-terminal end, such as nuclear localization signal (NLS), are related to subcellular localization and functional specialization (Brive et al., 2001). Further studies have shown that BAGs act as a molecular switch for multiple targets to maintain metabolic homeostasis. BAG family proteins are widely involved in a variety of biological processes, like apoptosis, tumor formation, stress response, neural differentiation, cell cycle, and so on (Behl, 2016).

Compared with the extensive and in-depth research on BAGs in animals, the research on BAG family in plants is relatively less, and mainly focuses on the model plant Arabidopsis. In Arabidopsis, seven AtBAGs have been found (Yan et al., 2003; Doukhanina et al., 2006; Nawkar et al., 2017). With the progress of plant genome research, a lot of BAGs have been found in the plant genome database. However, detailed studies of this family have only be done in *Arabidopsis thaliana*, *Musa acuminata*, *Oryza sativa*, *Solanum lycopersicum*, and *Zea mays* (summarized in **Table S1**). BAGs play a key role in plant growth, autophagy, and stress stimuli response (Thanthrige et al., 2020). Accumulated evidence shows that the mechanism of plant BAG actions is similar to those of animal BAGs. However, some member sequences of plant BAG proteins contain functional regions that are not found in animals, such as calcitonin junctions (IQ, CaM binding motif), which also reflects the diversity and specificity of plant BAGs in the exercise of functions (Kabbage and Dickman, 2008; Li et al., 2016a). The field of research on the unique functions of plant BAGs is still fragmentary.

Thanthrige et al. (2020) summarized the research progress of Arabidopsis BAGs, and emphasized their roles in regulating plant programmed cell death (PCD). Recently, there have been more studies on the function of BAGs in a variety of plants (summarized in **Table 1**). For example, the involvement of tomato BAGs in fruit ripening, environmental stress, and dark-induced leaf senescence was reported (Ding et al., 2020; He et al., 2021; Irfan et al., 2021; Ding et al., 2022; Jiang et al., 2022). The evolution of rice BAGs and their roles in cell death were studied (Bansal et al., 2022). Here, we focus on the major advances of plant BAGs on their structures, biological functions, and molecular mechanisms of plant growth and stress response, in order to provide valuable information for further directions in this field and important cues for crop bioengineering.

**Table 1:** The functional studies of plant BAGs in plant development and stress tolerance.

Species	Gene	Subcellular localization	Reference	Function	Reference
Arabidopsis	*AtBAG1*	Cytoplasm	Lee et al. (2016)	Growth; Salt	Lee et al. (2016)
	*AtBAG2*	Cytoplasm	Lee et al. (2016)	ABA; Drought; Heat	Arif et al. (2021)
	*AtBAG4*	Cytoplasm; Nucleus; ER	Lee et al. (2016); Locascio et al. (2019)	K^+^ transport; Stomatal movement; Salt; Cold; *Botrytis cinerea*	Doukhanina et al. (2006); Kabbage and Dickman (2008); Hoang et al. (2015); Locascio et al. (2019)
	*AtBAG5*	Mitochondria;	Li et al. (2016b)	Leaf senescence	Cui et al. (2015); Li et al. (2016a)
	*AtBAG6*	Vacuolar; Nucleus	Li and Dickman (2016); Fu et al. (2019)	Heat; Autophagy; Fungal resistance; ABA; Drought; Heat	Kang et al. (2006); Li et al. (2016b); Li and Dickman (2016); Nawkar et al. (2017); Fu et al. (2019); Arif et al. (2021)
	*AtBAG7*	Nucleus; ER	Williams et al. (2010); Lee et al. (2016); Li et al. (2017)	*Phytophthora capsica*; PlAMV; Heat; Cold	Williams et al. (2010); Li et al. (2017); Gayral et al. (2020); Zhou et al. (2021)
Soybean	*GmBAG7a*	ER	Zhou et al. (2021)	*Phytophthora capsici*	Zhou et al. (2021)
	*GmBAG6-1*			Cell death	Wang et al. (2020b)
Banana	*MusaBAG1*			*Fusarium oxysporum* f. sp. cubense	Ghag et al. (2014)
Rice	*OsBAG4*	Cytoplasm; Nucleus	Wang et al. (2020a)	Salt; Plant innate immunity and broad-spectrum disease resistance	You et al. (2016); Wang et al. (2020a)
Tomato	*SlBAG2;9 SlBAG5b*	Cytoplasm; Nucleus	He et al. (2021); Ding et al. (2022)	Dark-induced leaf senescence; Heat; Drought	Ding et al. (2020, Ding et al., 2022); He et al. (2021); Jiang et al. (2022)
Wheat	*TaBAG; TaBAG2*	Cytoplasm; Nucleus	Ge et al. (2016)	Heat; Salt	Ge et al. (2016)
Grape	*HSG1*			Heat; Flowering	Kobayashi et al. (2012)

### Protein structure of plant BAGs

The C-terminal of BAG family proteins contain at least one BAG domain, which is composed of three antiparallel bundles of alpha-helices containing 70~80 amino acids, and the second and third α helix contains highly conserved amino acid residues (Briknarová et al., 2001). BAG has been identified as a NEF chaperone family, which contains a BAG domain. This domain interacts with HSP70/HSC70 on its ATPase domain, and affects the nucleotide exchange by helping ATP combine with HSP70/HSC70 and release ADP, thus enhancing the quality control of protein. This way, the BAG family may establish a connection between the HSP70/HSC70 partner system and its substrate (Behl, 2016).

**Figure 1:**
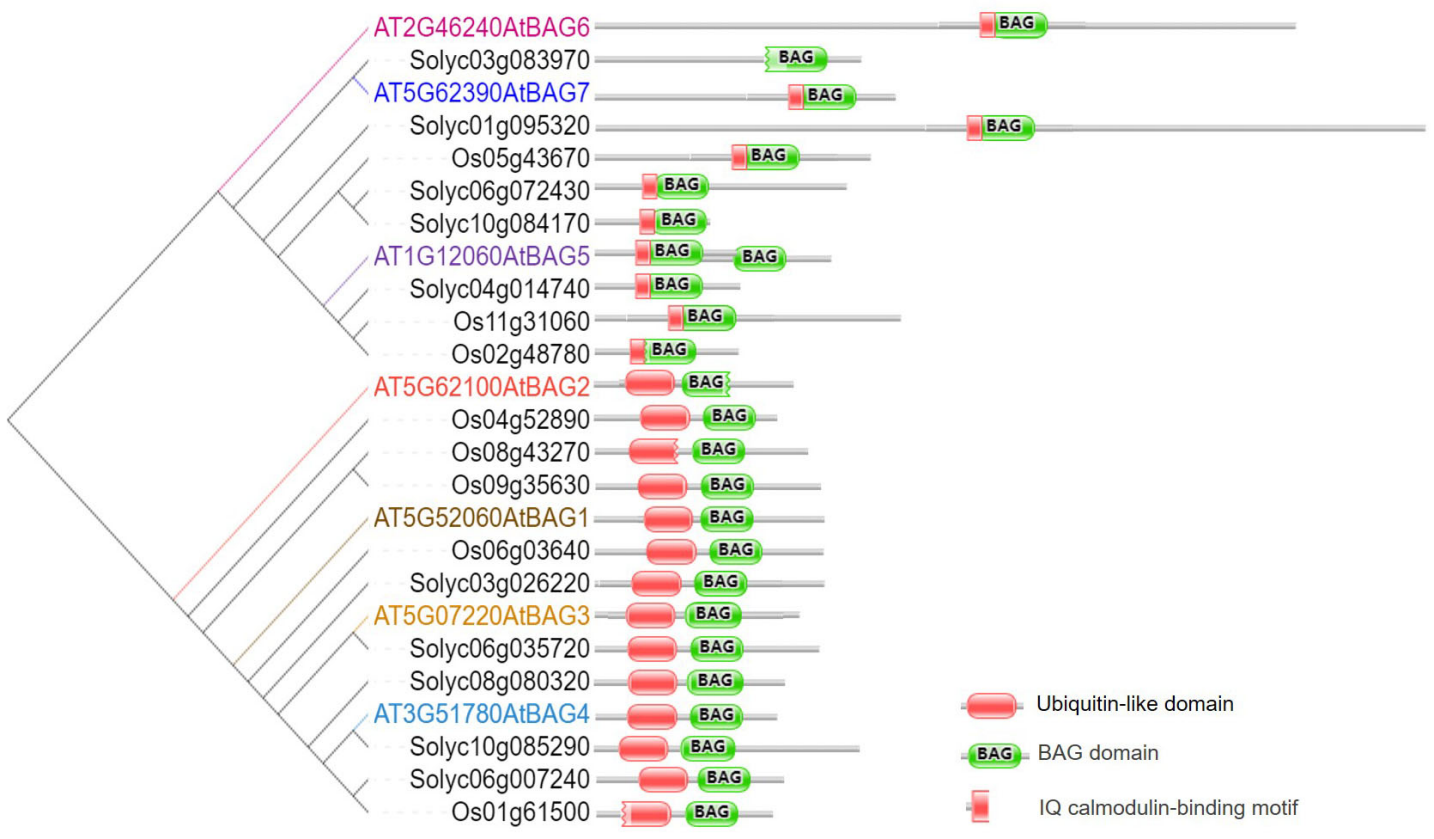
BAG domains of three plant species. Left: Phylogenetic tree of three plant species (At, *Arabidopsis thaliana*; Sl, *Solanum lycopersicum*; Os, *Oryza sativa*). The protein sequences of these plant species were generated using the PhyML in MEGA 7.0. Based on the phylogenetic data, these proteins are classified into two distinct sub-groups. Right: BAG domains were generated through pfam (http://pfam.xfam.org/). The legends of BAG domain, Ubiquitin-like domain, and IQ motif are listed on the right.

**Figure 2:**
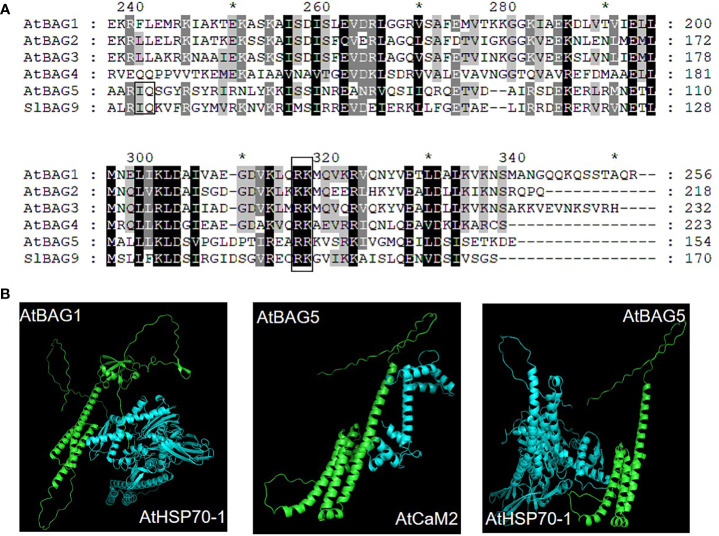
Recognition of AtBAG by Hsc70 and CaM. **(A)** Sequence alignment of AtBAG1-AtBAG5 and SlBAG9. Conserved residues are colored. Key residues responsible for the association of the BAG domain with Hsc70 and CaM are framed. **(B)** The models of the simultaneous interaction of AtBAG1 with AtHSP70 and AtBAG5 with AtCaM2 and the AtHSP70. Spatial structures of the AtBAG1, AtBAG5, AtHSP70, and, AtCaM2 were predicted based on target-template alignment on SWISS MODEL server (https://swissmodel.expasy.org/). Protein–protein docking models were generated using HADDOCK (https://wenmr.science.uu.nl/haddock2.4). * means the starting point of every 10 amino acid residues.

In plants, BAG protein family can be classified into two groups according to their structural characteristics through genome-wide alignment and conservative domain identification (Safder et al., 2022). The first group has a ubiquitin-like (UBL) domain at the N-terminal end, similar to the structural composition of animal BAG1. The second group defines plant-specific CaM-binding IQ motif near BAG domain (Yan et al., 2003). The first group of Arabidopsis includes four members, AtBAG1, AtBAG2, AtBAG3, and AtBAG4. Rice and tomato have five members each (**Figure 1**). They are homologous to animal BAG1, and have similar structure and function. Fang et al. (2013) determined the crystal structure of the AtBAG1–4 domains, indicating that they have a high degree of homology. Furthermore, the binding of the BAG domain of AtBAG1 to the nucleotide-binding domain (NBD) caused the Hsc70-NBD conformation to change to the open state and reduced the affinity of NBD for ADP, suggesting that AtBAGs act as a nucleotide-exchange factors for Hsp70/Hsc70 in Arabidopsis (Fang et al., 2013). AtBAG1 residues R220 and K221 make salt bridges with several Hsc70 acidic residues. The sequence aligning AtBAG1 and AtBAG5 showed that the R131 and R132 residues may be responsible for the association between AtBAG5 and Hsc70. Mutation of R131 and R132 to serine eliminated the linking between AtBAG5 and Hsc70 (Li et al., 2016a), indicating that the two acidic residues (R/K/; R/K) are important binding sites (**Figure 2**; **Figure S1**). According to the secondary structure alignment, the BAG domain of all Arabidopsis BAG proteins seems to be the short variant, which is found in animals and fungi, and so they may be presumably more ancient. The long BAG domains are restricted to vertebrates and nematodes (Doukhanina et al., 2006).

The second group of CaM-binding IQ motif in Arabidopsis contains AtBAG5, AtBAG6, and AtBAG7. Also, there are three and five members in rice and tomato, respectively (**Figure 1**). It is well known that CaMs interact with many kinases, and can transmit cellular signals carried by calcium ions, thus affecting morphogenesis, signal transduction, stress response, and other processes of plant cells (Zeng et al., 2015). While IQ motif can bind CaM and affect CaM and target proteins, suggesting a potential role in the plant Ca^2+^ signaling. The IQ motif of ATBAG6 is essential for the physical interaction of AtBAG6 with AtCaM to formation of specific complexes (Kang et al., 2006). Furthermore, CaM has two domains, N-lobe and C-lobe each has two calcium-binding sites. The CaM N-lobe presents the classic closed conformation (closed N-lobe) without Ca^2+^ (Ca^2+^-free), which named apo-CaM, while the C-lobe appears a semi-open conformation. And there are many interaction sites between amino acid positions 49~153 of the AtBAG5 structural crystal (AtBAG5-long) and the closed N-lobe of CaM. AtBAG5 and apo-CaM interaction is mainly through the recognition of IQ motifs by the semi-open C-lobe of CaM. Furthermore, Ca^2+^-free and Hsc70 each independently binds AtBAG5, while Ca^2+^-saturated CaM binds ATBAG5 and Hsc70 binds AtBAG5 in negative synergy. AtBAG5 acts as a signaling center using IQ and BAG domains to link Ca^2+^-signaling with Hsc70 partners to regulate plant senescence (Li et al., 2016a).

### Subcellular distribution of plant BAGs

BAGs have a diverse subcellular localization, including the cytosol, nucleus, mitochondria, vacuole, or endoplasmic reticulum (ER) (**Table 1**). Widespread distribution of BAGs in plants suggests that they play significant roles in numerous signal transduction pathways. Lee et al. (2016) detected the subcellular localization of AtBAG proteins by GFP fusion method. AtBAG1-3 protein localization is mainly in the cytoplasm, while AtBAG4 localization is mainly in the cytoplasm and nucleus. The nuclear localization of AtBAG4 has been further determined by immunostaining with anti-t7 antibody. In rice, the GFP-tagged OsBAG4, an AtBAG4 homologous protein, was co-transformed with the signal–red fluorescent protein into rice protoplasts and OsBAG4-GFP is mainly localized in the nucleus, while a small part in the cytoplasm (Wang et al., 2020a). In tomato, SlBAG1/SlBAG2/SlBAG8 belonging to the same family as Arabidopsis AtBAG1-4 were observed in the nucleus and also in the cytoplasm of the cell periphery (Irfan et al., 2021). Interestingly, SlBAG4 (homologous with AtBAG4) is expressed as intracellular and can represent the Golgi or ER (Irfan et al., 2021). Earlier, the ER localization of AtBAG4 has also been reported. AtBAG4 interacts with KAT1, facilitating KAT1 to reach the plasma membrane, to improve KAT1-mediated K^+^ transport (Locascio et al., 2019). KAT1-BAG4 interaction may occur at the ER exit site (ERES) and BAG4 may regulate this step when KAT1 moves out of the ER to the plasma membrane by the secretory pathway.

Though GFP-AtBAG5 and GFP-AtBAG6 are present as punctate spots, and the identity has not been known (Lee et al., 2016), however, AtBAG5 is localized in mitochondria (Li et al., 2016b) and AtBAG6 is in vacuole or nucleus (Li and Dickman, 2016; Fu et al., 2019). AtBAG5 with a mitochondrial target peptide is a mitochondrial localization protein, which is involved in the regulation of Ca^2+^ level (Li et al., 2016a; Fu et al., 2017). This feature is puzzling because of the central role of mitochondria in the intrinsic apoptotic pathway, but no animal BAGs is localized to the mitochondria. Interestingly, the tomato homologous protein SlBAG9 is not located in the mitochondria, but in the cytoplasm and nucleus (Ding et al., 2022). There is some controversy about the localization of AtBAG6. Co-transformation of YFP-fused AtBAG6 with RFP-tagged vacuolar markers into protoplasts revealed that YFP-ATBAG6 merged with vacuolar signal, indicating the relevant location for ATBAG in plant vacuoles, which was consistent with the occurrence of autophagy (Li and Dickman, 2016). However, the concentrated AtBAG6 in the purified nuclear fraction, but BAG6 was not detected in the purified tonoplast fraction. In addition, the green fluorescence of the BAG6-GFP fusion was distributed mainly in the nucleus. The transformation of onion epidermal cells further proved that BAG6 might be localized in the nucleus (Fu et al., 2019). In rice, the homologous protein OInBAG6 is located in the cytosol and nucleus (Bansal et al., 2022). In tomato, SlBAG2 and SlBAG5b reside on the cell membrane and are located in the nucleus (He et al., 2021). The localization of AtBAG6 and its homologous proteins remains to be elucidated. AtBAG7 is located in the ER and nucleus (Williams et al., 2010; Lee et al., 2016; Li et al., 2017). AtBAG7 is constitutively located in ER, acting as a part of the UPR (unfolded protein response) to regulate cell death pathways during heat stress (Williams et al., 2010). Under heat stress, AtBAG7 is activated by sumoylation and subjected to proteolytic treatment to transfer it from the ER to the nucleus, and then interacts with WRKY29 (Li et al., 2017). Plants can also recognize a culture filtrate (CF) stimulation, thereby releasing AtBAG7 from ER into nucleus. Subsequently, BAG7 localizes in the nucleus and actively regulates plant tolerance to Phytophthora through activating ERSI (Zhou et al., 2021). In soybean, GmBAG7a is similar to the Arabidopsis homologous gene in terms of *Phytophthora capsici* resistance. CF similarly induces GmBAG7a release from ER and nucleus translocation. Collectively, BAG7 localization and function are likely to remain unchanged in plant species (Zhou et al., 2021).

### BAGs in plant growth and development

The widespread presence of BAGs in plants indicates their importance to some extent. Accumulated studies have indeed shown that BAGs play important and diverse roles in many fields, including plant growth and development (summarized in **Table 1**). The rosettes of 4-5-week-old *bag2-1* plants are significantly larger than that of WT (Fang et al., 2013). An appropriate level of *AtBAG1* is essential for the normal growth of plants. The growth of 2-week-old *AtBAG1*-overexpressing plants are smaller than that of empty vector plants, showing a delay in growth (Lee et al., 2016). EBR1 (enhanced blight and blast resistance 1) encodes a previously unknown RING-type E3, which interacts with a BAG family protein OsBAG4, leading to its ubiquitination and degradation. So OsBAG4 can accumulate in *ebr1* mutant (You et al., 2016). The growth of *ebr1* and *OsBAG4*-overexpressing plants is significantly delayed, while *EBR1* overexpression promotes plant growth. Many genes associated with hormone pathways are regulated in *ebr*1 and *OsBAG4-*overexpressing plants. OsBAG4 is also supposed to affect certain hormone-mediated pathways (You et al., 2016). Therefore, OsBAG4-triggered autoimmunity must be fine-tuned to avoid stunted growth of WT rice (You et al., 2016). Besides these, there are also progresses in stomatal movement, leaf senescence, and flowering regulation.

#### Stomatal movement

Stomatal movement regulates photosynthesis and transpiration in plant, playing a key role in plant growth (Blatt et al., 2022). In guard cells, regulation of ion flux is critical for stomatal movement (Misra et al., 2019). KAT1 (Potassium channel protein 1) is a major inward rectifier channel in guard cells, which mediates the influx of potassium (K^+^), leading to stomatal opening. Locascio et al. (2019) screened the KAT1 interactors in *Arabidopsis* and found that AtBAG4 interacted with KAT1 and functioned when KAT1 reached the plasma membrane. Heterologous expression of *AtBAG4* increases KAT1 activity in yeast and *Xenopus oocytes*. Knock-out or overexpression of *AtBAG4* alters stomatal opening kinetics, which is consistent with a physiological role in regulating K^+^ flux (Locascio et al., 2019). Therefore, AtBAG4 plays a role in the dynamic regulation of stomatal pore size by regulating the inward rectifying K^+^ channel of KAT1.

#### Leaf senescence

**Figure 3:**
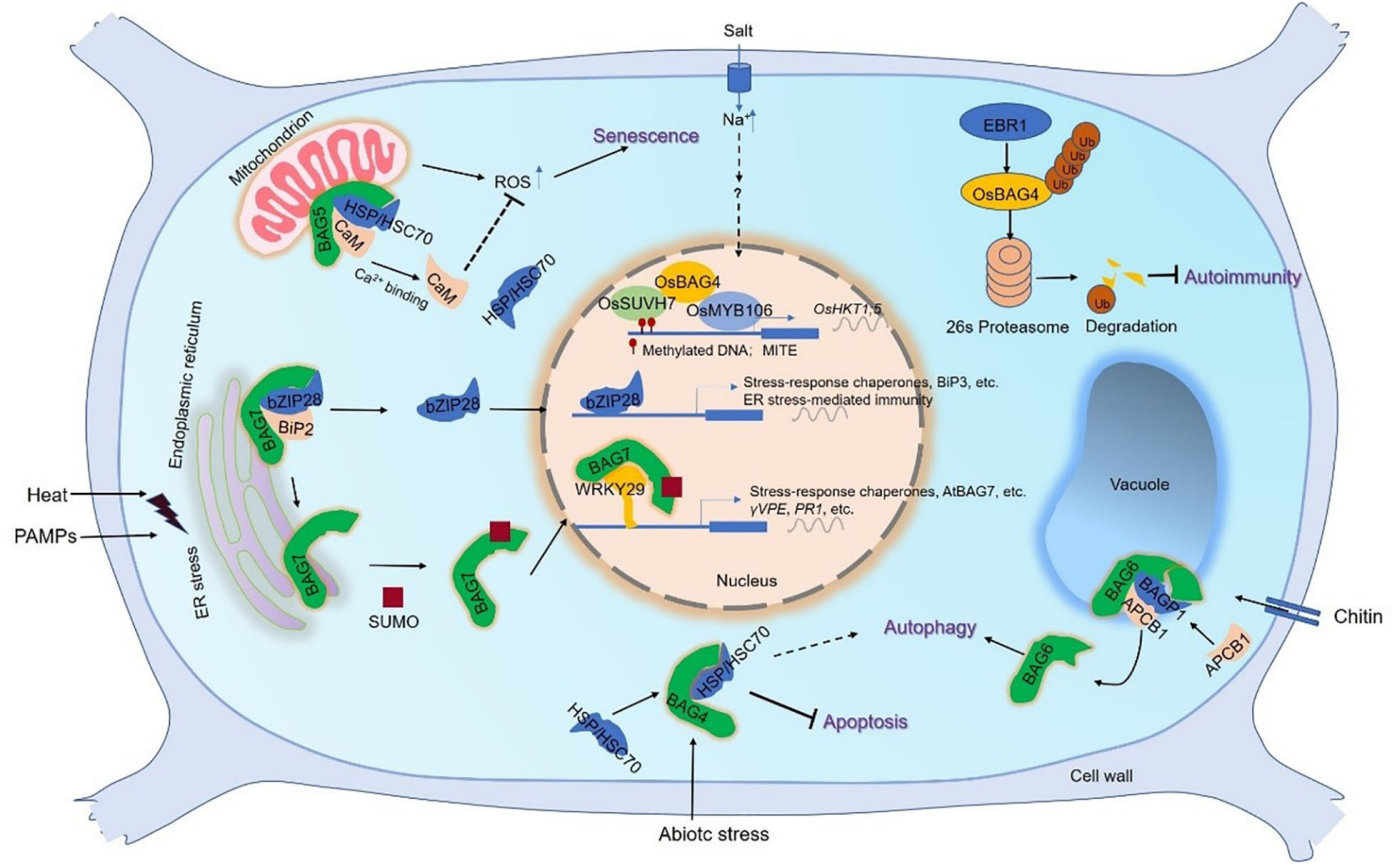
The working model of plant BAG-mediated signal pathway. The roles of BAG1–3 are less known. BAG4 plays a role in the cytoplasm, combines with HSP70/HSC70 chaperones, and is involved in the inhibition of cell death under abiotic stress (Hoang et al., 2015). Under salt stress, the rice OsBAG4 acts as a bridge between OsSUVH7 and OsMYB106 to promote the binding of OsMYB106 to MYB binding MYBE of OsHKT1;5 promoter, thereby activating *OsHKT1;5* expression to decreased salt sensitivity. OsSUVH7 reads the methylation of the MITE of OsHKT1;5 promoter (Wang et al., 2020a). The rice RING-type E3 ubiquitin ligase, EBR1, directly targets OsBAG4 for ubiquitination-mediated degradation (You et al., 2016). The mitochondrion-localized AtBAG5 forms a complex with CaM/HSC70 to regulate plant senescence. The complex promotes the production of ROS and accelerates leaf senescence at low Ca^2+^ levels. In contrast, the senescence is inhibited at high Ca^2+^ levels (Li et al., 2016a). BAG6 is involved in autophagy and is part of a fundamental defense pathway against necrotizing fungi. BAG6 interacts with BAGP1 and APCB1, both of which are necessary for BAG6 processing. BAG6 cleavage triggers autophagy in the host which (Li et al., 2016b). BAG7 mediated heat stress tolerance (Li et al., 2017). In ER, under normal conditions AtBAG7 binds AtBiP2 and AtbZIP28 transcription factors. Under heat stress conditions, unfolded proteins accumulate and AtBAG7 and AtbZIP28 dissociate from AtBiP2. Then AtBAG7 is sumoylated and proteolytically cleaved to translocate to the nucleus. Here, AtBAG7 interacts with WRKY29, which is able to induce the transcription of *AtBAG7* and other chaperone genes. Besides, AtbZIP28 enters the nucleus to induce the chaperone gene expression including AtBiP3. Similar to heat stress, ER stress is triggered when Phytophthora PAMPs are infected (Zhou et al., 2021). BAG7 binds to WRKY29 to regulate plant immunity mediated by ER stress, which is also directly activated by bZIP28. Thus, plants exhibit resistance to Phytophthora infection. Abbreviations: BAG, Bcl-2-associated athanogene; BAGP1, BAG-associated GRAM protein 1; BIP2, Binding immunoglobulin protein 2; bZIP28, basic leucine zipper 28; CaM, calmodulin; ER, endoplasmic reticulum; HSC70/HSP70, heat shock cognate 70/heat shock protein 70; MITE, miniature inverted repeat transposable element; ROS, reactive oxygen species; SUMO, small ubiquitin-like modifier; WRKY29, WRKY DNA-binding protein 29.

Senescence is the developmental PCD which results in the cell or tissue death of particular organ, occurring at the level of plant organs. Some genes, called senescence associated genes (SAG), precisely control the process of leaf senescence (Lim et al., 2007). Senescence is accelerated when BAG expression is inhibited, consistent with the notion that BAGs play a role in cell survival (Doukhanina et al., 2006). The *atbag4* mutant exhibits accelerates senescence phenotype, which is associated with PCD-related senescence. Under dark condition, the expression of *SlBAG2* and *SlBAG5b* is up-regulated and ROS is reduced, which further delays the leaf senescence process (He et al., 2021). During the dark period, compared with WT plants, the expression levels of chloroplast degradation-related genes and leaf senescence-related genes were significantly decreased in *BAG*-overexpressing plants. Therefore, it is suggested that tomato *SlBAG2* and *SlBAG5b* are involved in chloroplast degradation and leaf senescence by regulating the expression of related genes in addition to regulating the production of ROS (He et al., 2021). In the dark-induced senescence experiment, compared with the *bag5-1*/*bag5-2* plants, the gain of function showed an early senescence phenotype (Li et al., 2016a; Fu et al., 2017), with high ROS levels and upregulated *SAG* expression. A model of the effect of Ca^2+^ on AtBAG5 in leaf senescence has been proposed (**Figure 3**). AtBAG5 regulates leaf senescence through Hsc70 signaling pathway. Apo-CaM binds to the IQ motif on AtBAG5, but does not affect the binding of Hsc70 to the BAG domain. When Ca^2+^ concentration increases, CaM changes its interaction with AtBAG5. This unique binding pattern can further disrupt the binding of Hsc70 to the BAG domain, resulting in Hsc70 release. Free Hsc70 is associated with the reduction of PCD through the inhibition of the expansion of ROS. In conclusion, AtBAG5 plays a signaling hub function, connecting the Ca^2+^ signaling network and the Hsc70 chaperone system, thereby regulating plant senescence (Li et al., 2016a).

#### Flowering

Many signals promote the transformation of meristem from vegetative growth to reproductive growth, leading to early flowering. The *atbag4* and *atbag6* mutants showed early flowering and a branched inflorescence phenotype, suggesting that they play a role in plant development (Doukhanina et al., 2006). HSG1 was identified as a putative grape BAG protein (Kobayashi et al., 2010; Kobayashi et al., 2012). Overexpression of *HSG1* in Arabidopsis promoted the transformation of meristem from vegetative growth to reproductive growth through activation of the flowering promoter CONSTANS (CO) in the photoperiod pathway, resulting in early flowering (Kobayashi et al., 2012).

### BAGs in abiotic stress tolerance

Abiotic stress response is very important for sessile plants, because plants cannot survive unless they can cope with environmental changes. Understanding this response process is very important. So far, a series of abiotic stress response genes have been isolated and their functions have been accurately described in transgenic plants (Hirayama and Shinozaki, 2010; Kumar et al., 2022). BAGs widely mediate plant responses to various abiotic stresses, such as salt stress, drought stress, and extreme temperature stress (summarized in **Table 1**). For example, *AtBAG4* overexpression confers tobacco tolerance to cold, salt, UV, and oxidative stress (Doukhanina et al., 2006; Kabbage and Dickman, 2008).

#### Heat stress

Recently, extreme weather events such as heat stress often occur. Plant cells constantly sense and receive heat signals through altering physiological metabolism and activating various defense mechanisms, and maintain their life activities (Mo et al., 2021). *AtBAG4* gene expression is induced by heat stress (Nawkar et al., 2017). AtBAG4 functions in the cytoplasm and binds with HSP70 to suppress abiotic-stress-induced cell death (Doukhanina et al., 2006). Previous studies have shown that AtBAG5 is localized to the mitochondria and may be an important part of the mechanism regulating mitochondrial cell death (Li et al., 2016b). The heat-shock element in the AtBAG5 promoter suggests that it is responsive to heat induction (Nawkar et al., 2017). In our study, it has been found that HT induces *AtBAG5* expression and the T-DNA mutant *atbag5* exhibits increased basic thermotolerance (data not shown). Interestingly, in our previous study on the *SlBAG9* function, a homologous gene of *AtBAG5* in tomato, further proved that it negatively regulates heat tolerance (Ding et al., 2020; Ding et al., 2022; Jiang et al., 2022). HT (high temperature) induces high expression levels of *SlBAG9* at the transcriptional and protein levels, and *SlBAG9*-overexpressing plants of Arabidopsis and tomato show high sensitivity to HT stress (Ding et al., 2020; Ding et al., 2022). *SlBAG9* promoter can drive *GUS* gene expression in tobacco leaves under normal or HT condition, and the HSE1 is essential for HT-induced GUS activity (Ding et al., 2022).

AtBAG6 is a heat-inducible protein (Kang et al., 2006; Kabbage and Dickman, 2008; Li et al., 2017; Fu et al., 2019). Mutation in the *AtBAG6* gene can enhance the basic thermotolerance of Arabidopsis (Echevarría-Zomeño et al., 2016). However, *bag6* single mutation does not respond to acquired thermotolerance, but knockout of *AtBAG6* significantly improves the heat tolerance of *fes1a* (a NEF that increases molecular chaperone efficiency that essential for heat tolerance in Arabidopsis) mutant. AtBAG6 functions as an inhibitor of Fes1A (Fu et al., 2019). Additional knock-down of *AtBAG6* in *fes1a* increases the expression of HSP70 and small HSP 17.7. IQ motif is an essential domain for BAG6. Thermotolerance evaluation shows that overexpression of BAG6–mIQ has no effect on the thermotolerance of the *fes1abag6* double mutant, while BAG6 protein in the *fes1abag6 double* mutant results in a decrease of thermotolerance. It is further considered that knockdown of *AtBAG6* may release CaM or CaM-like proteins from the nucleus, which in turn alters the dynamics of nuclear calcium signaling to enhance HSP expression in *fes1abag6* and improve acquired thermotolerance. Fu et al. (2019) further showed that a single mutation of *AtBAG1/2/3/4/6* had no effect on plant acquired thermotolerance. Nawkar et al. (2017) also confirmed the heat inducibility of *AtBAG6* gene, but unlike other studies, the deletion of *AtBAG6* enhanced the sensitivity of plants to heat stress according to the basic thermotolerance test and electrolyte leakage test. In Arabidopsis heat stress experiment, the survival rates of *bag2* and *bag6* single mutants and *bag2bag6* double mutants were lower. Compared with WT seedlings, mutants exhibited higher ROS content after heat stress (Arif et al., 2021). The heat sensitivity of *atbag6* mutants might not be attributed to defects in autophagy, as heat stress-induced autophagy is not regulated by AtBAG6 (Li et al., 2016b).

AtBAG7 is localized to the ER and participates in the maintenance of the ER stress pathway, which is a unique role of plant BAG proteins. AtBAG7 translocates from the ER to the nucleus in response to HT and regulates the UPR pathway (Williams et al., 2010). Thermotolerance of AtBAG7 depends on proteolytic cleavage and translocation in addition to sumoylation (Li et al., 2017). The model of AtBAG7-mediated heat tolerance was proposed (**Figure 3**): In ER, under normal conditions AtBAG7 binds AtBiP2 and AtbZIP28 transcription factors. Under heat stress conditions, unfolded proteins accumulate and AtBAG7 and AtbZIP28 dissociate from AtBiP2. Then AtBAG7 is sumoylated and proteolytically cleaved to translocate to the nucleus. Here, AtBAG7 interacts with WRKY29, which is able to induce the transcription of *AtBAG7* and other chaperone genes. Besides, AtbZIP28 enters the nucleus to induce the chaperone gene expression including AtBiP3 (Li et al., 2017).

In addition to Arabidopsis, heat-induced BAG is also found in other plants. In maize, 13 BAG family gene expressions are induced by heat stress (Hu et al., 2013). In grape, a putative grape BAG HSG1 is identified as a heat shock inducible protein. Arabidopsis plants overexpressing *HSG1* shows heat tolerance even at extremely high temperature (Kobayashi et al., 2010). In wheat, *TaBAG2* expression is induced by heat stress and *TaBAG2* overexpression increases Arabidopsis thermotolerance (Ge et al., 2016). Interestingly, Arabidopsis co-overexpressing *TaBAG2* and *TaHSP70* exhibits better thermotolerance compared to single transformed plants. The thermotolerance of *TaHSP70*-overexpressing Arabidopsis plants is not prominent. It is possible that excess TaHSP70 might bind to heat shock factor (HSF), which in turn reduces its combination with heat shock element sequence, thereby reducing the expression of heat shock gene and protein expression (Liu et al., 2003). Therefore, when *TaHsp70* and *TaBAG2* are co-overexpressed in plants, the excessive TaBAG2 can bind to excess TaHsp70 protein to release HSF, thereby increasing thermotolerance in plants (Ge et al., 2016).

#### Cold stress

Cold tolerance tests of plants overexpressing *AtBAG4* shows that dead patches are apparent on WT leaves after cold stress, but leaves with low *AtBAG4* expression remains intact (Doukhanina et al., 2006; Kabbage and Dickman, 2008). AtBAG4 appears to protect plants from cold stress by inhibiting PCD (Doukhanina et al., 2006). Kawamura and Uemura (2003) found that the content of a range of plasma membrane proteins, including AtBAG7, undergoes major increase during the early stages of cold acclimation. ER stress is induced by cold treatment in WT and *atbag7* mutants (Williams et al., 2010). AtBAG7 responds to cold stress in cells undergoing ER stress by delaying the PCD pathway. This result is consistent with the data related to AtBAG4 during cold stress (Williams et al., 2010).

#### Salinity stress

For plants, high salinity is the most popular abiotic stresses. In Arabidopsis, *AtBAG4* expression is induced by salt (Nawkar et al., 2017). Overexpression of *AtBAG4* increases salt tolerance in Arabidopsis and rice (Doukhanina et al., 2006; Hoang et al., 2015). Transgenic rice seedlings overexpressing *AtBAG4* have many characteristics of salt tolerant rice varieties under salt stress. Heterologous *AtBAG4* expression in rice might significantly improve the salt tolerance of rice by maintaining the redox state (Hoang et al., 2015). The expression of Hsp70 and AtBAG4 facilitates protein folding and prevented protein denaturation in a high ROS condition to maintain the efficiency of cellular processes and mitigate ROS production. Salt alone significantly inhibits *AtBAG6* and *AtBAG7* expression, but the addition of ACC (1-Aminocyclopropanecarboxylic Acid) to salt solution can significantly reactivate the expression of *AtBAG6* and *AtBAG7*, which plays a crucial role in inhibiting salt-induced cell death (Pan et al., 2016).

In wheat, salt stress induces *TaBAG* gene expression, but the response of *TaBAG* gene to heat stress is not obvious. Arabidopsis overexpressing *TaBAG* and *TaBAG2* increases salt tolerance (Ge et al., 2016). Recent evidence suggests that rice *OsBAG4* has a positive effect on salt stress tolerance (Wang et al., 2020a). *osbag4-1* mutants have salt sensitive phenotype such as reduced survival rates and elevated shoot Na^+^/K^+^ ratios. OsBAG4 acts as a bridge between OsSUVH7 and OsMYB106 to promote the binding of OsMYB106 to MYB binding cis-element (MYBE) of *OsHKT1;5* promoter, thereby activating *OsHKT1;5* expression (Wang et al., 2020a).

#### Drought stress

*AtBAG2* and *AtBAG6* are negatively associated with drought stress (Arif et al., 2021). The sensitivity of seed germination of *bag2bag6* double mutants and their single mutants to ABA was lower than that of WT. The survival rate of mutants is higher than that of WT under drought treatment. Compared with WT, these mutants display differential transcription levels of stress-related genes such as *RD29A* and *RD29B*, ABA biosynthesis gene *NCED3*, and ABA response gene *ABI4* (Arif et al., 2021). Recently, *SlBAG9* overexpression in Arabidopsis increased the sensitivity to drought stress. The reduced tolerance may be due to *SlBAG9*-mediated down-regulation of stress-related gene expression and severe oxidative damage. Overexpression of *SlBAG9* under drought significantly suppressed some stress-related genes like *ABI3*, *RD29A*, *DREB2A*, and *P5CS1* (Jiang et al., 2022).

### BAGs in biotic stress tolerance

Plants are frequently faced with biotic stresses such as microbial pathogens and insects. To combat biotic stress, plants have evolved complex organismal defense mechanisms (Gimenez et al., 2018). In Arabidopsis, when evaluating the knockout of the seven homologues of the *BAG* family, it is noted that the *atbag6* knockout lines, but not other knockout lines, exhibits a susceptibility phenotype when injured by the necrotrophic fungus *Botrytis cinerea*, suggesting that *AtBAG6* may be associated with basal resistance (Doukhanina et al., 2006). In addition, with the application of defense related signals such as SA and MeJA, the expression level of *AtBAG6* increases, supporting its participation in the host defense mechanism. From these findings, it is considered that *A*tBAG6 may play a role in basal tolerance through limiting disease development of *Botrytis cinerea* (Doukhanina et al., 2006; Kabbage and Dickman, 2008; Nawkar et al., 2017). It has been reported that AtBAG6 activation mediated by aspartyl protease cleavage is necessary for triggering autophagy and resistance to fungal pathogen (Kabbage et al., 2016; Lee et al., 2016; Li et al., 2016b). It is confirmed that the AtBAG6 protein interacts with AtBAGP1 and AtAPCB1, respectively, to form complexes, and participate together in antifungal infection (Li and Dickman, 2016). AtBAG6 is processed at a single caspase 1-like cleavage site by binding to protein partners including AtBAGP1 and AtAPCB1 (**Figure 3**). AtBAG6 cleavage requires AtBAGP1 and AtAPCB1, which are necessary for subsequent host resistance. Knockdown of *AtBAGP1* or *AtAPCB1* results in blockade of AtBAG6 function (Li and Dickman, 2016). Expression of AtBAG6 mutated at the cleavage site results in inhibition of autophagy and unhindered fungal growth. In all, these findings demonstrate the AtBAG6 function involved in plant PCD and indicate that AtBAG6-induced cell death not only to kill individual cells, but to rescue whole plants (Nawkar et al., 2017). Recently, AtBAG7 has been shown to be associated with the local accumulation of *Plantago asiatica* mosaic virus, but not with *Turnip mosaic* virus (Gayral et al., 2020). AtBAG7 was elucidated to have dual roles in the ER-to-nucleus pathway and in Arabidopsis and soybean responses to the hemibiotrophic oomycete pathogen *P. capsici.* (Zhou et al., 2021). Specifically, it functions as a sensitizer in the ER, but acts as an anti-Phytophthora in the nucleus. The ER-to-nucleus translocation of AtBAG7 is triggered by Phytophthora infections (**Figure 3**), which is similar to HT stress; however, this process can be hindered with PsAvh262-mediated BiP accumulation.

In addition to Arabidopsis, BAGs were also found to participate in biological stress in some other plants. In rice, OsBAG4 is necessary to trigger PCD and is sufficient to improve rice tolerance to *Xanthomonas oryzae* pv. oryzae and *Magnaporthe oryzae* (You et al., 2016). Plants overexpressing *OsBAG4* exhibited autoimmunity, increased disease resistance, and stunted growth. Among OsBAGs, OsBAG4 is involved in plant innate immunity by directly linking with EBR1, which is a RING-type E3 ligase, directly targets OsBAG4 for ubiquitination-mediated degradation (**Figure 3**). OsBAG4 accumulation in rice is sufficient to trigger PCD and improve resistance to pathogenic infection. Accumulation of *OsBAG4* in *ebr1* or overexpression of *OsBAG4* triggered autoimmunity (You et al., 2016). Interestingly, the AtBAG4, an OsBAG4 homolog, appeared to protect plants from abiotic stresses, with no effect on disease tolerance (Doukhanina et al., 2006; Hoang et al., 2015). The roles of BAG proteins in PCD and monocotyledonous and dicotyledonous immunity may be evolutionarily different (You et al., 2016). In soybean, the specific induction of the soybean *GmBAG6-1* is found in resistant lines whereas almost no expression of this gene is detected in sensitive soybean lines responsive to soybean cyst nematode (SCN) (Kandoth et al., 2011). Most remarkably, however, *GmBAG6-1* induction in NIL-R is observed to be significantly reduced during infection by a virulent SCN population. *GmBAG6-1* induces cell death in yeast, as does *AtBAG6* in soybean. As part of its strategy to overcome soybean defense responses, the virulent SCN can target GmBAG6-1 (Wang et al., 2020b). In banana, *MusaBAG1*-overexpression enhances resistance to Foc (fungus *Fusarium oxysporum* f. sp. cubense (Foc) (Ghag et al., 2014). This is the first time to develop transgenic bananas with high tolerance to FOC by using native genes like *MusaBAG1*.

## Conclusions and perspectives

In animals, BAG family members are widely involved in tumor regulation, cell apoptosis, stress response, and other biological processes, so that their biological functions have been widely concerned and studied. In plants, BAG family proteins participate in biological processes such as plant PCD and autophagy, and have certain functional conservatism in plant responses to stresses (summarized in **Table 1**). However, there are still many outstanding issues: (1) In the past decade, a burst of new plant genome sequences has been published, more BAG families in plant species will be excavated, and the function of different BAGs still need to be further explored. (2) At present, the research of BAG family in plants mainly focuses on the model plant *Arabidopsis thaliana*, and the study on the function of BAG family in other species is relatively few. In many plants with important scientific research and economic value, the research involving BAG protein family still stays at the level of structural comparison. Little is known about the regulatory network of BAGs in plant cells. (3) The presence of the CaM-binding domain in some BAGs is unique to plants, indicating a potential role for BAGs in Ca^2+^/CaM signaling. However, the function of specific CaM-mediated by IQ domain of BAGs is still unclear. (4) BAGs are involved in multiple plant PCD pathways. What specific signaling pathways need to be revealed? (5) The physical basis property of BAGs has been well-established. However, the mechanisms of action such as interaction with other proteins and post-translational modification need to be further studied in detail. Although some protein partners/interactors have been found (summarized in **Table S2**; **Figure 1**), the structural or physical basis of BAG interaction is basically unknown for the newly identified signal components. With availability of massive genomic and proteomic data and cutting-edge research technologies and methods, it is hoped that more plant researchers will take up this challenge and test for BAG function and mechanism, especially those newly reported and typical BAGs. The in-depth study of BAG family members can provide some research ideas for the improvement of agronomic traits such as enhancing crop resistance, delaying crop aging, and improving crop yield.

## Author contributions

HD and HJ conceived and designed the review. HJ, XL, and XW analyzed the data. HJ, YW, and PX contributed to literature collection. HD, QX, and HD contributed to create graphics and editing. HD and HJ wrote the manuscript. All authors contributed to the article and approved the submitted version.
