# Supplementary material for: Functional insights of plant bcl-2–associated ahanogene (BAG) proteins: Multi-taskers in diverse cellular signal transduction pathways
Source: Front Plant Sci. 2023 Mar 28;14:1136873. doi: 10.3389/fpls.2023.1136873 (PMC10086319; doi:10.3389/fpls.2023.1136873)
Supplement: Supplementary file 1 [file DataSheet_1.docx]

Supplementary Material

**Functional Insights of Plant Bcl-2–associated Ahanogene (BAG) proteins: Multi-Taskers in Diverse Cellular Signal Transduction Pathways**

***Hailong Jiang^1^, Xiaoya Liu^1^, Peixiang Xiao^1^, Yan Wang^1^, Qihui Xie^1^, Xiaoxia Wu^1,2^, Haidong Ding^1,2*^***

*Correspondence author

E-mail address: Haidong Ding (hdding@yzu.edu.cn), ORCID ID: 0000-0003-0644-238X

# Supplementary Tables

**Table S1 Compilation of BAG multigene family identified from plant genomes**

| **Specie(s)** | **Gene numbers** | **Developmental and stress response** | **Reference** |
| --- | --- | --- | --- |
| Arabidopsis | 7 | Cold; Osmotic; Salt; Drought; Oxidative; UV-B; Wounding; Heat; Hormones; Pathogen attack | Yan et al. (2003); Doukhanina et al. (2006); Nawkar et al. (2017) |
| Banana | 13 | Salt; PEG; *Fusarium oxysporum* | Dash and Ghag (2022) |
| Rice | 6/7/8 | Heavy metal; *Magnaporthe oryzae*; Hormones; Brown planthopper; Heat; Drought | Rana et al. (2012); Zhou et al. (2021b); Bansal et al. (2022) |
| Tomato | 10/11 | Mannitol; Salt; Cold; Heat; UV; Hormones | He et al. (2021); Irfan et al. (2021); Jiang et al. (2022) |
| Maize | 13 | Heat | Hu et al. (2013) |

**Table S2 Summary of plant BAG interacting proteins**

| **Species** | **Name** | **Interacting protein** | **Method** | **Reference** |
| --- | --- | --- | --- | --- |
| Arabidopsis | AtBAG1 | Hsc70-4 | Co-IP | Lee et al. (2016) |
|  | AtBAG4 | KAT1; HSC70-4 | BiFC; Co-IP | Doukhanina et al. (2006); Locascio et al. (2019) |
|  | AtBAG5 | CaM; HSC70 | Isothermal titration calorimetry (ITC) | Li et al. (2016a) |
|  | AtBAG6 | AtAPCB1; AtBAGP1 | Y2H; Co-IP | Li and Dickman (2016) |
|  | AtBAG7 | WRKY29; AtbZIP28; AtBiP2; SUMO | Y2H; Co-IP | Li et al. (2017) |
| Soybean | GmBAG6-1 | Hg-VAP2; 7E05 | Y2H | Wang et al. (2020b) |
| Rice | OsBAG4 | OsMYB106 | Co-IP; LUC/REN; Pull-down | Wang et al. (2020a) |
| Wheat | TaBAG2 | TaHsp70; TaCaMs | Y2H; BiFC | Ge et al. (2016) |

# Supplementary Figures


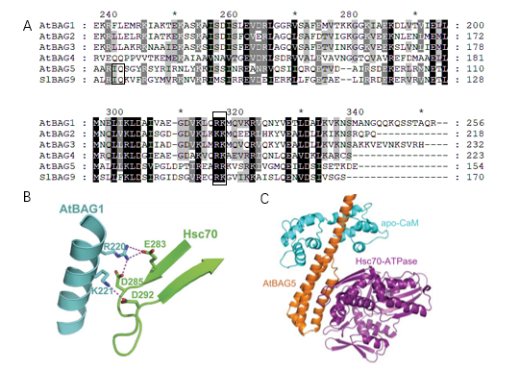


**Supplementary Figure** S1 Recognition of AtBAG by Hsc70 and CaM. (A) Sequence alignment of AtBAG1-AtBAG5 and SlBAG9. (B) Detailed simulation of the interaction of AtBAG1 (cyan) with Hsc70 (green). (C) A model of the simultaneous interaction of AtBAG5 with apo-CaM and the Hsc70 ATPase domain. The BAG domains from the AtBAG5-long/apo-CaM and AtBAG1/Hsc70complexes are superposed to simulate the interaction of AtBAG5 with apo-CaM andHsc70. The data is from Li (Li et al. 2016a).

References

Bansal, R., Kumawat, S., Dhiman, P., Sudhakaran, S., Rana, N., Jaswal, R., et al. (2023). Evolution of bcl-2 anthogenes (BAG) as the regulators of cell death in wild and cultivated Oryza species. *J. Plant Growth Regul*. 42, 348-364. doi: 10.1007/s00344-021-10552-7

Dash, A., and Ghag, S. B. (2022). Genome-wide in silico characterization and stress Q17 induced expression analysis of BcL-2 associated athanogene (BAG) family in musa spp. *Sci. Rep*. 12, 625. doi: 10.1038/s41598-021-04707-5

Doukhanina, E. V., Chen, S., van der Zalm, E., Godzik, A., Reed, J., and Dickman, M.B. (2006). Identification and functional characterization of the BAG protein family in Arabidopsis thaliana. *J. Biol. Chem*. 281, 18793–18801. doi: 10.1074/jbc.M511794200

Ge, S., Kang, Z., Li, Y., Zhang, F., Shen, Y., Ge, R., et al. (2016). Cloning and function analysis of BAG family genes in wheat. *Funct. Plant Biol*. 43, 393–402. doi: 10.1071/FP15317

He, M., Wang, Y., Jahan, M. S., Liu, W., Raziq, A., Sun, J., et al. (2021). Characterization of SlBAG genes from Solanum lycopersicum and its function in response to dark-induced leaf senescence. *Plants (Basel)*. 10, 947. doi: 10.3390/plants10050947

Hu, L. Z., Chen, J. T., Guo, J. J., Zhao, Y. F., Zhu, L. Y., and Huang, Y. Q. (2013). Functional divergence and evolutionary dynamics of BAG gene family in maize (Zea mays). *Inter. J. Agric. Biol*. 15, 200–206.

Irfan, M., Kumar, P., Ahmad, I., and Datta, A. (2021). Unraveling the role of tomato bcl-2-associated athanogene (BAG) proteins during abiotic stress response and fruit ripening. *Sci. Rep*. 11, 21734. doi: 10.1038/s41598-021-01185-7

Jiang, H., Ji, Y., Sheng, J., Wang, Y., Liu, X., Xiao, P., et al. (2022). Genome-wide identification of the bcl-2 associated athanogene (BAG) gene family in Solanum lycopersicum and the functional role of SlBAG9 in response to osmotic stress. *Antioxidants* 11, 598. doi: 10.3390/antiox11030598

Lee, D. W., Kim, S. J., Oh, Y. J., Choi, B., Lee, J., and Hwang, I. (2016). Arabidopsis BAG1 functions as a cofactor in Hsc70-mediated proteasomal degradation of unimported plastid proteins. *Mol. Plant* 9, 1428–1431. doi: 10.1016/j.molp.2016.06.005

Li, L., Xing, Y., Chang, D., Fang, S., Cui, B., Li, Q., et al. (2016a). CaM/BAG5/Hsc70 signaling complex dynamically regulates leaf senescence. *Sci. Rep*. 6, 31889. doi: 10.1038/srep31889

Li, Y., and Dickman, M. (2016). Processing of AtBAG6 triggers autophagy and fungal resistance. *Plant Signal. Behav*. 11, e1175699. doi: 10.1080/15592324.2016.1175699

Li, Y., Williams, B., and Dickman, M. (2017). Arabidopsis b-cell lymphoma2 (Bcl-2)-associated athanogene 7 (BAG7)-mediated heat tolerance requires translocation,sumoylation and binding to WRKY29. New Phytol. 214, 695–705. doi: 10.1111/nph.14388

Locascio, A., Marqués, M. C., García-Martínez, G., Corratgé-Faillie, C., Andrés-Colás, N., Rubio, L., et al. (2019). BCL2-ASSOCIATED ATHANOGENE4 regulates the KAT1 potassium channel and controls stomatal movement. Plant Physiol. 181, 1277–1294. doi: 10.1104/pp.19.00224

Nawkar, G. M., Maibam, P., Park, J. H., Woo, S. G., Kim, C. Y., Lee, S. Y., et al. (2017). In silico study on arabidopsis BAG gene expression in response to environmental stresses. Protoplasma 254, 409–421. doi: 10.1007/s00709-016-0961-3

Rana, R. M., Dong, S., Ali, Z., Khan, A. I., and Zhang, H. S. (2012). Identification and characterization of the bcl-2-associated athanogene (BAG) protein family in rice. Afr. J.Biotechnol. 11, 88–99. doi: 10.5897/AJB11.3474

Wang, J., Nan, N., Li, N., Liu, Y., Wang, T. J., Hwang, I., et al. (2020a). A DNA methylation reader-chaperone regulator-transcription factor complex activates OsHKT1;5 expression during salinity stress. Plant Cell. 32, 3535–3558. doi: 10.1105/tpc.20.00301

Wang, J., Yeckel, G., Kandoth, P. K., Wasala, L., Hussey, R. S., Davis, E. L., et al. (2020b). Targeted suppression of soybean BAG6-induced cell death in yeast by soybean cyst nematode effectors. Mol. Plant Pathol. 21, 1227–1239. doi: 10.1111/mpp.12970

Yan, J., He, C., and Zhang, H. (2003). The BAG-family proteins in Arabidopsis thaliana. Plant Sci. 165, 1–7. doi: 10.1016/S0168-9452(03)00121-3

Zhou, H., Li, J., Liu, X., Wei, X., He, Z., Hu, L., et al. (2021b). The divergent roles of the rice bcl-2 associated athanogene (BAG) genes in plant development and environmental responses. Plants (Basel). 10, 2169. doi: 10.3390/plants10102169
